# Sensorial, textural, and rheological analysis of novel pistachio‐based chocolate formulations by quantitative descriptive analysis

**DOI:** 10.1002/fsn3.3637

**Published:** 2023-08-23

**Authors:** Morad Mousazadeh, Mohammad Mousavi, Zahra Emam‐Djomeh, Salar Ali Ahmed, Mehri Hadinezhad, Hamed Hassanzadeh

**Affiliations:** ^1^ Department of Food Science, Engineering and Technology, Faculty of Agricultural Engineering and Technology University of Tehran Karaj Iran; ^2^ Food Technology Department, College of Agricultural Engineering Sciences Salahaddin University‐Erbil Erbil Iraq; ^3^ Ottawa Research and Development Center Agriculture and Agri‐Food Canada (AAFC) Carling Avenue Ottawa Ontario Canada; ^4^ Department of Food Science and Hygiene, Faculty of Para‐veterinary Ilam University Ilam Iran

**Keywords:** pistachio oil spread, principal component analysis, sensory evaluation, texture attributes

## Abstract

Principal component analysis (PCA) was used to investigate the effects of pistachio oil (7.5 and 15%), xanthan gum (0 and 0.3%), distillated monoglyceride (0.5 and 1%), and cocoa butter (7.5 and 15%) on the sensorial descriptors of spread based on pistachio oil. The response variables were the most significant spread texture attributes: hardness, graininess, meltability, adhesiveness to spoon, adhesiveness to mouth, spreadability, fluidity, and oiliness. PCA revealed that the first two principal components explained 90% or more of the variance between the data. The first principal component was dominated by the descriptors' adhesiveness and hardness on the positive side and the descriptors' oiliness and fluidness on the negative side. The descriptor spreadability had a high positive loading on the second principal component. Herschel–Balkley and power law models were fitted to confirm the sensory evaluation results on different formulations. In the current research, the power law model seemed to be more accurate for fitting the samples. In terms of the selected texture attributes determined by the sensory evaluation, using component plot, the optimum combination of variables was found as follows: 15 pistachio oil, 7.5% cocoa butter, 0.3% xanthan gum, and 1% distilled monoglyceride that produced desirable spreads that mimic commercial spread.

## Graphical abstract

Principal component analysis (PCA) was used to investigate the effects of pistachio oil (7.5 and 15%), xanthan gum (0 and 0.3%), distilled monoglyceride (0.5 and 1%), and cocoa butter (7.5 and 15%) on the sensorial descriptors of spread based on pistachio oil.

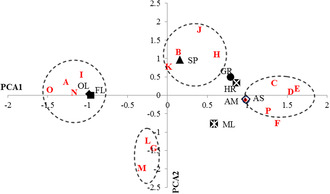

## 1 INTRODUCTION

Pistachio nuts are a good source of protein, lipid, and essential fatty acids for human nutrition (D'Evoli et al., 2015) (Table 1) and are a common snack rich in nutrients such as magnesium, thiamin, potassium, fibers, phytosterols, and vitamin B6 (De, 2020). Pistachio nuts (*Pistacia vera L*.), as a special nut, mainly contain more than 55% oil, with high proportions of polyunsaturated and monounsaturated fats, which are associated with improving cholesterol levels and promoting heart health. Oleic acid is the predominant fatty acid of pistachio oil (PO) with 56%–64% (Mousazadeh et al., 2013). This oil also contains essential fatty acids in the human diet such as linoleic and linolenic (Boualem et al., 2022; Shakerardekani et al., 2015; Yang et al., 2009). Pistachios are commonly consumed as whole nuts (raw, roasted, or salted) or used as ingredients in a variety of processed foods, including spreads, confections, and bakery products. Pistachio oil can be used for many purposes including cooking, salad dressing, and flavoring. In addition to food applications, pistachio oil is also used as a component of some moisturizers and skin cosmetic products (Alasalvar & Shahidi, 2008). There is some proof that sea transportation or storage in importing countries causes aflatoxin contamination of pistachio nut during the export process (Cheraghali & Yazdanpanah, 2010). The nuts cannot be used by the consumers if the total amount of mycotoxins in nuts (especially aflatoxin B1) exceeds the permissible limit (Cheraghali & Yazdanpanah, 2010; Shakerardekani et al., 2012; Soares Mateus et al., 2021; Vahedi & Mousazadeh, 2016). The increasing growth of new products made from nuts (such as nut chocolate) and the use of suitable packaging materials can reduce the loss of this product, especially due to contamination with mycotoxins. The application of nuts potentially in food use can introduce it to consumers as a healthier and nonanimal breakfast snack. Spreads are semi‐solid foods that contain different levels of fat. The spread is classified as water/oil (W/O) emulsion that must be stabilized by different emulsifiers and gums (Liu et al., 2021). Unhealthy and high saturated fat diets can be replaced by spreads that have been fortified via functional ingredients such as pistachio oil and pistachio paste. Spreads generally have been consumed by different societies so the spread can be very valuable in their diets. Because of functional factors, a positive relationship existed between a high intake of pistachio products and a low risk of cancer in some organs (Yahia et al., 2019). Functional foods can provide useful components in the human diet. As the functional food market continues to grow and due to the functional properties of pistachios, results show that consumers are willing to include pistachios in food (Aryee & Boye, 2014; Tomaino et al., 2010). The mentioned functional properties of pistachio make pistachio paste and pistachio oil good complementary for spreads to provide a functional food product for everyone specially growing child (González‐Tomás & Costell, 2006; Tomaino et al., 2010). Chocolate is a water/oil (W/O) emulsion model with various formulations including fat (cocoa butter [CB]), water, salt, emulsifiers, stabilizers, antioxidants, etc. (Khaleghi Yazdi et al., 2021; Tanaka et al., 2009). One of the important components of chocolate production is fat. Fat adds shortening, richness, and tenderness to the product and improves mouth feel, taste, and perception (Pareyt et al., 2009). Some studies have determined that partial replacement of consumed fat with vegetable oils can improve spreadability at refrigerated temperature (4°C) and nutritional values such as favorable fatty acid profiles and also lower cholesterol levels in chocolate products with a mixture of vegetable oils and butter (Bemer et al., 2016; Kim et al., 2005). On the other hand, chocolate is an emulsion system that must be stabilized. Currently, there are a significant number of polysaccharide biopolymers such as xanthan gum (XG) and emulsifiers such as distillated monoglyceride (DMG) which are used to create the emulsion phase structure of these food systems (Mousazadeh et al., 2013). Food texture is a general term that encompasses several related physical properties, indicating that instrumental analysis cannot fully represent the overall texture experience. It is important to derive and identify objective measurements that show a high correlation with sensory attributes that are of interest to the processing industry and consumers. Sensory evaluation is defined as a scientific and practical method that calculates, analyzes, and interprets responses to products through the senses of sight, smell, touch, taste, and sound (Sharif et al., 2017). Sensory evaluation is connected to exactness, accuracy, and sensitivity and avoiding false‐opposite results (Hough & Garitta, 2012). For an accurate sensory evaluation, the analyst must ensure the correct targeting of the test, the selection of the appropriate experimental design, the appropriate method of sample preparation and delivery, and also the correct analysis of the data. Also, a sensory tester should always consider the correctness of the applied method and the errors related to the test environment in each stage of the tests. Finally, the relationship between the structure of food and the concept of its texture should be considered (Gharibzahedi, Mousavi, Hamedi, & Ghasemlou, 2012; Radočaj et al., 2011). Although texture evaluation via a texture analyzer such as Instron provides informative data, the sensory evaluation definitely needs to confirm a new food and its inclusion in diet society. The textural characteristics of spreads such as hardness (HR), graininess (GR), meltability (ML), adhesiveness to spoon (AS), adhesiveness to mouth (AM), spreadability (SP), fluidness (FL), and oiliness (OL) commonly play a vital role in consumer appeal, buying assessments and eventual consumption (Beckett, 1999, 2000a; Bonczar et al., 2002). Also, optimizing different formulas can be a suitable solution to strengthen research and development activities on new or existing products. Optimization for the production of PO‐based chocolates is very crucial due to the favorable textural characteristics, the design of functional devices and equipment, and the control of the production process, maintenance, and shelf life (Gharibzahedi et al., 2011). Although several studies have been reported texture analysis using both sensorial and mechanical evaluation (Deshpande et al., 2005; Morzel et al., 2000; Shakerardekani et al., 2013; Veland & Torrissen, 1999) of nut chocolate, little information is available on the correlation between sensory and instrumental measurements, and no study has been specifically targeted at the correlation of sensory evaluation of chocolate texture based on pistachio oil. Various researches on spreads have shown that most of them show non‐Newtonian characteristics, so they can be evaluated using different models such as Bingham, Herschel–Bulkley, Power law, and Casson (Gonçalves & Lannes, 2010). Non‐Newtonian fluids have unstable conditions during the process, and the process components can have a great impact on their structural characteristics (Mewis, 1979). To the best of our knowledge, detailed investigations of textural characteristics of PO‐based chocolates and optimization of their structural components have not been available. In our previous research, the rheological properties of pistachio oil spread were investigated (Mousazadeh et al., 2013). The objective of the present study was to evaluate the texture of novel functional chocolate based on PO as a function of various practical formulations and the acceptability of different formulation spreads based on pistachio oil and factors affecting their quality by sensory evaluation.

**TABLE 1:** Nutritional composition of pistachio nut (per 100 g) (D'Evoli et al., 2015)

Nutrient	Pistachio
Calories (kcal)	557
Protein (g)	21
Total fat (g)	44
Saturated (g)	5
Monounsaturated (g)	23
Polyunsaturated (g)	13
Carbohydrate (g)	28
Dietary fiber (g)	10

## 2 MATERIALS AND METHODS

### 2.1 Chemicals and materials

Pistachios were procured from the local market of Rafsanjan (Iran) in November 2019 and were manually cracked and flaked and then crushed in a disk mill (Glen Mills, Clifton, NJ, USA). A screw press (Model NB 90, Kimiagaran Products Co., Kerman, Iran) was used for the oil expression. The profile of the fatty acids of the oil prepared in this research was as follows (mol %): C14:0 (0.6%), C16:0 (11%), C16:1 (0.7%), C18:0 (1.7%), C18:1 (59.5%), C18:2 (25%), C18:3 (0.2%), C20:0 (0.3%), and C20:1 (0.6%) as detected by gas chromatography of methyl esters. Ingredients such as sugar, milk powder, CB, and salt powder were purchased from Gorji Biscuit Co (Tehran, Iran). Xanthan gum (XG) and locust bean gum (LBG) (LBG is purified carob seed endosperm [*Ceratonia siliqua*]) were prepared by Sigma‐Aldrich Co. PGPR 4175, PGPR 4110, distilled monoglycerides (DMG 0291), and DMG0295 were purchased from Emulsion‐Holland B.V., Zierikzee, Holland. Polyglycerol polyricinoleate (PGPR) is also a vital ingredient for chocolate production, which is obtained from polycondensation of castor oil and glycerol. It is a diverse mixture that has a polyglycerol component and is dominated by di‐, tri‐, and tetraglycerol. The manufacturer has suggested DMG0295 and PGPR4175 for formulations with less than 20% oil and DMG0291 and PGPR4110 for chocolate formulations with more than 20% oil.

### 2.2 Chocolate preparation

Chocolates were prepared similarly to the method presented by Mousazadeh et al. (2013). The details were as follows: a mixture of water‐soluble mixture of sugar (45%, w/w), milk powder (13.5%, w/w), pistachio paste (10%, w/w), XG (0 and 0.3%, w/w), LBG (0.09%, w/w), and salt (0.04%, w/w) was refined to particle size <30 μm using multirolls refiners. PO (7.5 and 15%, w/w), surfactants (DMG; 0.5 and 1%, w/w), and PGPR (0.3%, w/w) were separately mixed as fat‐soluble ingredients, then mixed with the former mixture at 50°C temperature. Then, the obtained mixture was refined for 2 hours using a laboratory‐scale conch. The conching process caused physicochemical changes in the texture of mixture and final product as chocolate was achieved (Mousazadeh et al., 2013). Sixteen PO‐based chocolates (A‐P samples) with different formulations were prepared according to the full factorial design (Table 2). Preliminary experiments determined that increasing the studied concentration levels in the chocolate formula caused favorable textural changes in PO‐based chocolate. Optimum values of 0.09% and 0.3% w/w for LBG and PGPR were determined, respectively (Imram, 1999). Other ingredients such as pistachio paste and milk powder were added to the amount recommended by the company.

**TABLE 2:** The combination of formulations based on different levels of independent variable.

Sample coding	PO (% w/w)	CB (% w/w)	XG (% w/w)	DMG (% w/w)
A	15	7.5	0	0.5
B	7.5	7.5	0.3	0.5
C	7.5	15	0	1
D	15	15	0.3	0.5
E	7.5	15	0.3	0.5
F	7.5	7.5	0.3	1
G	7.5	15	0.3	1
H	15	7.5	0.3	1
I	15	7.5	0.3	0.5
J	7.5	7.5	0	0.5
K	15	7.5	0	1
L	7.5	15	0	0.5
M	15	15	0.3	1
N	15	15	0	1
O	15	15	0	0.5
P	7.5	7.5	0	1

Abbreviations: CB, cocoa butter; DMG, distilled monoglyceride; PO, pistachio oil; XG, xanthan gum.

### 2.3 Sensory analysis

According to the method of Szczesniak (González‐Tomás & Costell, 2006; Tanaka et al., 2009), among 30 volunteers for sensory analysis from the University of Tehran's students, 15 panelists (consisting of 7 females and 8 males, aged between 22 and 45 years) were selected. Panelists were trained to become familiar with the characteristics of the texture of spreads and enrich their ability to assess the sensory attributes and scaling procedure in 2‐h sessions prior to evaluation. Hardness (HR), graininess (GR), meltability (ML), adhesiveness to spoon (AS), adhesiveness to mouth (AM), spreadability (SP), fluidness (FL), and oiliness (OL) were judged. HR was evaluated by each panel member by placing a sample that was taken out of the refrigerator in the mouth, between the molar teeth, and biting down equally, measuring the maximum force required to compress the food. GR was evaluated by placing a sample in the mouth and chewing a few times and finally was rated. For evaluating ML, the panelists put a piece of sample in their mouth and graded melting rate. AS was measured by putting a spoon in the spread and bringing it out slowly. AM was rated by the panel by pressing each sample to the palate with the tongue. The sample was spread using a spoon on the peace of bread to evaluate SP. The panelists put the spoon into the spread container and turned it several times to evaluate the fluidity of the spread. OL was evaluated by placing a sample in the mouth and swallowing it; then, the aftertaste of each sample in terms of content of oil was rated. A 9‐point hedonic scale sensory test was used (9 like extremely/ high intensity, 1 dislike extremely/low intensity). All samples with three‐digit random numbers were given to panelists on a tray in individual partition. Orders of samples were completely randomized. Fifty‐gram samples in Petri dishes were presented to the panelists and they were asked to rinse their mouths with water between evaluations of each of samples. Samples were evaluated at ambient temperature.

### 2.4 Rheological analyses

Oscillatory shear measurements were performed by a Physical Rheometer MCR 301 (Anton Paar, GmbH, Ostfilden, Germany) and a four‐blade St14 vane. The vane was inserted into the cup vertically using the dimensions suggested by Steff (Steffe, 1996). The temperature was set at 25°C. The power law and Herschel–Bulkley models were fitted to the experimental data to find the optimal flow curves with the highest accuracy (Gharibzahedi et al., 2011; Vereecken et al., 2009):

$$\text{Power}\;\mathrm{law}\;\text{model}:б=k\;\left(\gamma^{n}\right).$$

$$\text{Herschel}–\text{Bulkley}:б={б}_{0}+k\;\left(\gamma^{n}\right).$$

where *б* is shear stress, *k* is consistency coefficient (*K*‐value), *γ* is shear rate, *n* value is flow behavior index, and *б*_0_ is yield stress.

### 2.5 Experimental design and statistical analysis

Principal component analysis (PCA) was used to transform a number of correlated features into a new group of principal components, which are linear arrangements of the main descriptors and are not correlated with each other. In this method, the number of main components and the number of primary descriptors are equal (Smith, 1988). Furthermore, they are classified in such a way that the change is applied to the dataset by successive reductions in the principal component. Usually, most of the variance in the data and the distribution of all descriptors and independent variables are expressed by the first two components, and for this reason, PCA is known as a dimensionality reduction technique (Smith, 1988). In the current research, the studied factors included the concentration of emulsifying agents (DMG) and stabilizing agents (XG), PO, and CB. Finally, data correlation analysis was performed using Pearson test in SPSS 13 software (SPSS Inc., USA).

## 3 RESULTS AND DISCUSSION

### 3.1 Original descriptors in PCA

**FIGURE 1:**
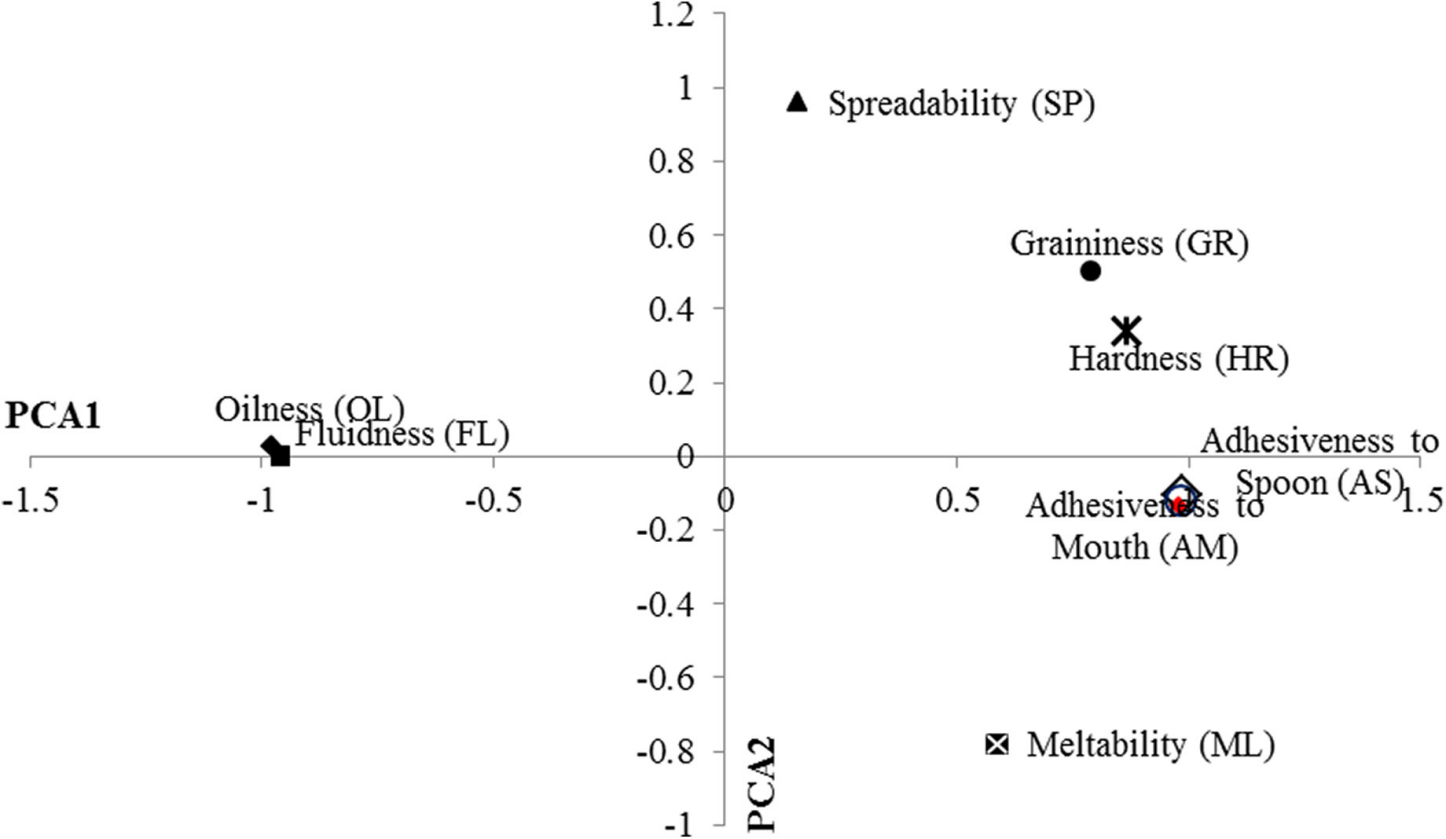
Loadings of sensory descriptors on the first and second principal components (PC).

PCA was applied to the combined data of chocolate formulations and different descriptors. As can be seen in Table 3, the first two principal components together include more than 93% of the data changes for each of the studied descriptors (the first principal component alone expresses 72.5% of the changes). In previous research, different sensory attributes have been introduced to describe the same primary product characteristics such as hardness, stickiness, stickiness, moisture content, and particle size. In the current research, quantitative descriptive analysis (QDA) was used to identify those descriptors that, according to the perception of the evaluators, were identified as the most important in evaluating the texture of pistachio oil (Stone & Sidel, 1993). Attributes HR and GR were used for evaluating different formulations at refrigerator temperature (4–7°C) and the others were characterized at ambient temperature (25°C). FL, AS, and SP belong to appearance attributes, whereas the rest belong to mouthfeel attributes. As observed in Figure 1, for the first principal component, descriptors HR, GR, AS, and AM had high positive loadings, but on the other hand, descriptors OL and FL had high negative loadings. High positive loading for SP descriptor and negative loadings for ML are observed in the second principal component. (Figure 1). A high and significant correlation (*p* < .01, Table 4) was found among sensorial attributes in highly negative loading side of PCA1 (HR, GR, AS, and AM), and also for descriptors at highly positive loading side (OL and FL). So, it could be possible to estimate correlation by component plots. The results also demonstrated that sensory descriptors had comparable loadings for the first two principal components. It indicates that PCA test can be used to evaluate different formulations with high predictability. Data analysis and sensory evaluation by PCA recognized the interrelationships between the descriptors and variables that have a significantly effect on them. The first principal component was characterized by nonadhesive/adhesive features on the negative and positive sides, respectively (Figure 1). An adhesive texture is associated with AS, AM, and GR mouthfeel, while nonadhesive texture is associated with OL mouthfeel and FL appearance (Figure 1). The differences between these two texture types are answerable for most of the variety that is found in different formulations that have been developed by original variables (PO, XG, DMG, and CB). Apart from some correlations with GR and ML, SP had a very low correlation with all other descriptors (Figure 1). Radočaj et al. (2011) and Kim et al. (2005) also found no significant relationship between AD and OL, but in the classification system used by RSM, AD and HR are both used as descriptors for different formulas.

**TABLE 3:** Components for describing variation in PCA test.

Component	Initial eigenvalues			Extraction sums of squared loadings		
	Total	% of variance	Cumulative %	Total	% of variance	Cumulative %
1	6.521	72.451	72.541	6.521	72.451	72.541
2	1.938	21.539	93.990	1.938	21.539	93.990
3	0.247	2.744	96.734			
4	0.140	1.559	98.293			
5	0.091	1.008	99.301			
6	0.035	0.387	99.687			
7	0.016	0.181	99.868			
8	0.009	0.097	99.965			
9	0.003	0.035	100.000			

**TABLE 4:** Pearson's correlation coefficients between sensorial attributes.

Correlations	AS	FL	SP	GR	ML	AM	OL	HR
AS	1	−0.961**	0.062	0.705**	0.645**	0.995**	−0.985**	0.785**
		0.000	0.819	0.002	0.007	0.000	0.000	0.000
FL		1	−0.159	−0.716**	−0.528*	−0.952**	0.960**	−0.780**
			0.555	0.002	0.036	0.000	0.000	0.000
SP			1	0.556*	−0.639**	0.041	−0.156	0.603*
				0.026	0.008	0.881	0.563	0.013
GR				1	0.098	0.713**	−0.704**	0.851**
					0.717	0.002	0.002	0.000
ML					1	0.655**	−0.578*	0.254
						0.006	0.017	0.343
AM						1	−0.974**	0.789**
							0.000	0.000
OL							1	−0.807**
								0.000
HR								1

* Significant at *p* < .05

** Significant at *p* < .01.

### 3.2 Correlation between responses

**FIGURE 2:**
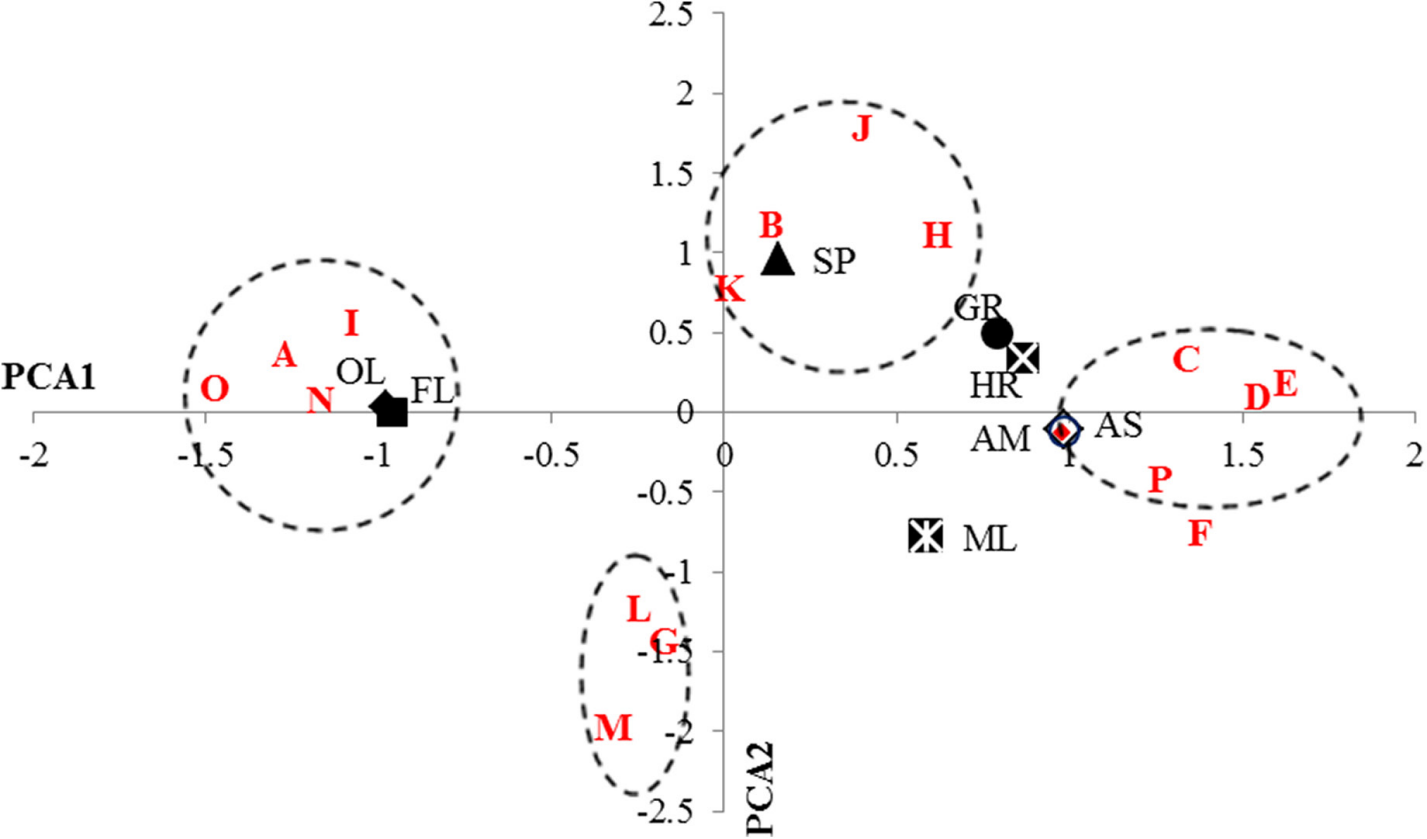
Different formulations and sensorial attributes plot derived from principal component analysis sample scores averaged over assessors.

Table 4 shows the correlation coefficients between product responses. All texture characteristics except FL and OL were positively correlated with HR, and as a result, softer samples have higher fluidity and will be oily. Because of positive correlation between HR and SP, semisolid foods such as the spread via firmer structure are more spreadable (Table 4). Based on the results, SP can be used to separate formulations that have close adhesive/nonadhesive properties. For example, samples C, D, E, F, and P have similar characteristics with respect to adhesion but can be distinguished based on SP. Also, samples A, I, O, and N have common characteristics with regards to OL, but they can be distinguished by SP and ML (Figure 2). Based on the obtained results from the PCA plot (Figure 2), the studied samples can be grouped into four classes based on the descriptors SP, OL/FL, and adhesiveness: group 1: spreadable: B, H, J, and K; group 2: oily/lubricants: A, I, O, and N; group 3: adhesive: C, D, F, and P; group 4: moderately melt; and oily/lubricant: G, L, and M.

### 3.3 Correlation between descriptors and original variables

Much research has been done to study the correlation between chocolate components and texture properties to provide justification for different texture types. In the present study, a significant correlation was detected between original variable content and the texture descriptors that are dominant on two first principal components (Table 5). The obtained correlation shows well that the value of the variables is the most effective feature for chocolate texture prediction because the first principal component explains most of the chocolate texture variance. As observed in Table 5, PO and XG content have noticeable significant effect on HR (*p* < .01). The negative correlation between PO and HR indicates that an increase in PO concentration leads to a decrease in HR. It can be justified by the high concentration of monounsaturated (oleic) and polyunsaturated (linoleic) fatty acids as well as low SFC values at higher PO levels. The instrumental HR of dark chocolate decreased remarkably with increasing percentage of hazelnut oil due to changes in SFC values (Nattress et al., 2004). Full et al. (1996) also reported that there was a strong positive correlation between instrumental HR and SFC of chocolate spreads at 20°C. As expected, when the XG content was changed from 0 to 0.3 wt%, HR was increased which can be a result of hardening the texture of the spread by increasing the viscosity for samples containing high XG concentration (Table 5). In addition, the interactions of XG with LBG can increase consistency and HR due to the association of the double helical structure of XG with unsubstituted mannosyl residue sequences in galactomannans (Casas & Garcia‐Ochoa, 1999). Gharibzahedi et al. (2011) stated that increasing the concentration of XG reduced the size of the emulsion particles and also decreased the droplet size distribution. It appears that decreasing particle size increases consistency, and then spreads with smaller particle sizes have higher HR values compared to spreads with larger particle sizes. Narine and Marangoni (1999) had also previously reported that fat systems with a larger crystal size were often characterized by a lower firmness (Hinds et al., 1994; Yeh et al., 2003). According to Table 5, DMG and CB had no significant correlation with HR. Adhesiveness or stickiness is defined as the force required to overcome the attractive forces between the food surface and the surface with which the food sample comes in contact (Glibowski et al., 2008). Table 5 clearly shows that PO and CB had adverse significant effect on AS and AM, whereas CB and XG had no significant correlation on AS and AM. Radočaj et al. (2011) also found that HO content at higher levels decreased the spread's adhesiveness. Our result suggested that during the conching step, strong interactions were formed between solid particles such as sugar and CB, and PO mainly via XG and XG‐LBG as matrix‐forming agents. These internal linkages are stronger than interaction between samples and external surfaces resulting in a decrease in adhesiveness (Afoakwa et al., 2007; Casas & Garcia‐Ochoa, 1999). Samples C, D, E, F, and P were more adhesive than other (Figure 2). The correlation of PO, XG, DMG, and CB with descriptors FL and OL of spread have been shown in Table 5. PO content had synergism correlation via both FL and OL. Thus, increasing PO content caused increase in descriptors’ FL and OL. The viscosity of samples had been decreased due to the high concentrations of monounsaturated (oleic) and polyunsaturated (linoleic) fatty acids and lower SFC values at higher PO levels. It seems that decreasing viscosity increases fluidity of samples. According to the negative correlation of DMG and CB with FL, When DMG and CB content were increased, the FL was decreased. Our previous study (Mousazadeh et al., 2013, 2014) demonstrated that increase in DMG and CB content caused increase in apparent viscosity, and this can be related to the denser structure and smaller particles in the spread containing DMG, and the samples became thicker with the addition of DMG. Gharibzahedi, Mousavi, Khodaiyan, and Hamedi (2012) stated that the addition of emulsifier is able to cover the surface and causes the formation of a larger number of smaller particles and leads to a decrease in the mobility of molecules. CB has a specific texture due to unique interactions of polymorphic lipid structures. Brunello et al. (2003) reported that polymorphism, through its effects on material microstructure, dramatically affects tissue properties. It seems that the increase in CB concentration makes the interactions between the microstructural elements in the molecular arrangement of the spreads stronger, so the fluidity of the samples decreases. The addition of PO and CB significantly increased OL, while increasing the content of XG and DMG alone or in combination with each other had the opposite effect. Figure 2 represents that the formulations A, I, N, and O show more FL and OL than others. On the other hand, the mentioned samples contain a maximum amount of PO or CB and a minimum of XG or DMG (Table 2). In this regard, Mousazadeh et al. (2013) reported that increasing PO and CB content increased oiling out and fluidity while XG and DMG content decreased them. When PO content increased from 7.5% up to 15%, descriptor GR decreased while increasing the content of CB and XG (Table 5). It can be hypothesized that GR is directly related to the extent of fat crystallization and the effect of other variables such as CB and XG on crystal morphology, and this phenomenon, in turn, is the main cause of roughness in spreads. The reduction in GR through increasing PO content can be related to the low SFC of the spreads containing high levels of unsaturated fatty acids, indicating that the addition of XG greatly increased the formation of a network matrix and thus increased the grain size of the constituents of the samples. CB crystals are usually in the β2 form, which is the largest among the different crystal form of lipids. On the other hand, increasing the CB content decreased FL (Table 5), and FL and GR have a significant negative correlation (Table 4). Although the spread has fluid property, the characteristics of melting is one of the important properties of spreads during eating because of the presence of CB and DMG. According to Table 5, CB had a positive high correlation with ML, whereas the correlation of XG and DMG was negative. During the production of chocolate, one of the main steps is the crystallization of cocoa butter into a specific three‐clinic polymorphic form, which is called the V (or β2) form (Chu & Resurreccion, 1991, 2005; Wille & Lutton, 1966); it is reported that cocoa butter (CB) crystallized in the V form can provide good texture and stable melting characteristics in fine chocolates (Beckett, 2000b). Marangoni and Mcgauley (2003) showed that cocoa butter crystallization kinetics and nucleation rate were significantly related to fat crystal network and melting rate. It seems that the presence of XG and DMG prevents the formation of β2 crystals which caused reduction in melting rate. SP is the most important characteristic among sensorial attributes, and it is the first sign of quality of spread of the consumer perspective. As seen in Figure 1, SP could be used to separate different formulations via different texture types. PO content had adverse correlation with SP, whereas other variables had positive correlation. Spreads based on the suitable formulation can be spreadable in different regions where there is inadequate temperature control at higher ambient conditions. The significant effects of variables on texture and sensorial attributes indicate that the variables and level of them will have been chosen in this research. As the PO increased with decrease in XG, DMG, or CB in the samples, SP decreased. This confirms that the fat‐based semi‐solid spreads with higher liquid oil contents are also softer and more easily recovered after deformation. The predominant (about 80–85%) fats of PO are unsaturated fatty acids (Mousazadeh et al., 2014). Spreadable properties in spreads are due to saturated fatty acid so increasing PO content caused decrease in SP. On the other hand, SP and HR showed positive correlation (Table 4), so the factors that decreased viscosity and HR caused to decrease in SP. XG content had a significant positive linear effect (*p* < .001) on the spread's spreadability. The same result was obtained for DMG and CB content. At higher XG, DMG, and CB content, the higher values of SP show firmer spreads. From Table 4, it can be seen that the correlation between SP and HR is positive (*p* < .05). What is clear is that a more compact internal structure is formed, where all the liquid oil with the stabilizer is in a saturated fat matrix. Carbohydrates are incorporated, which can increase the strength of internal bonds. An increase in CB content led to an increase in SP (Table 5). This is likely a result of the higher percentage of saturated fatty acids in CB, which cross‐link with stabilizing crystals (compound of saturated oils) and produce a spreadable texture. PCA plots allow to researcher to recognize the samples that have excellent sensorial attributes. According to Figure 2, samples B, H, J, and K had the greatest potential to spread. Regards to the functional properties of PO, among those samples, the sample that has maximum of PO and minimum of CB are the best sample in terms of texture. So, samples H and K were selected for further research.

**TABLE 5:** Correlation coefficient between independent variables content and the eight sensory descriptors.

Variable	Descriptor							
	HR	GR	ML	AS	AM	SP	FL	OL
PO	^1^‐0.48** ^2^0.000	−0.46** 0.000	0.072 0.269	−0.330** 0.000	−0.358** 0.000	−0.533** 0.000	0.434** 0.000	0.362** 0.000
XG	0.209** 0.001	0.174** 0.007	−0.131* 0.042	0.080 0.217	0.059 0.359	0.264** 0.000	0.087 0.180	−0.138* 0.033
DMG	−0.117 0.071	−0.067 0.301	−0.163* 0.012	0.084 0.196	0.049 0.446	0.171** 0.008	−0.155* 0.016	−0.129* 0.046
CB	−0.004 0.951	0.192** 0.003	0.667** 0.000	−0.287** 0.000	−0.299** 0.000	0.557** 0.000	−0.245** 0.000	0.258** 0.000

^1^ Pearson correlation.

^2^ Sig. (two‐tailed).

* Significant at *p* < .05

** Significant at *p* < .01.

### 3.4 Rheological models

The best shear stress–shear rate plots were obtained based on two famous models: Power law and Herschel–Bulkley to approve the sensorial results. Based on Table 6, the two mentioned models were compared by determination coefficient (R^2^). Because of higher R^2^, the power law model was better when choosing to describe the textural behavior of spread compared to the Herschel–Bulkley. The highest *K*‐value was obtained for samples C, D, and E, whereas the least values belonged to samples A, I, and O. The droplet interactions turned to be stronger so the network has become stronger because of adding XG and CB to the spread formulation. On the other hand, PO addition caused decrease in *K*‐value. The DMG concentration had the least effect on Power law parameters. XG and CB showed negative effect on n value, whereas PO had positive effect on that. The results of PCA (Figure 2) were in the same direction as rheological results. The extracted results from tools could approve the sensorial results. Based on Figure 2, samples C, D, and E showed more structural characteristics and consistency, while samples A, I, and O were more fluid and showed a weaker internal structure.

**TABLE 6:** Power law and Herschel–Bulkley 493 model parameters for each formulation's flow curve.

Formulations	Model			
	Power law			Herschel–Bulkley
	K (Pa.s)^*n*^	n	*R* ^2^	*R* ^2^
A	9.04 ± 1.28^k^	0.84 ± 0.021^a^	0.999	0.653 ± 0.087
B	31.12 ± 1.71^i^	0.70 ± 0.024^c^	0.948	0.629 ± 0.102
C	451.07 ± 6.32^a^	0.31 ± 0.016^j^	0.981	0.474 ± 0.077
D	335.66 ± 3.32^b^	0.36 ± 0.024^i^	0.982	0.636 ± 0.065
E	325.76 ± 7.65^c^	0.38 ± 0.033^h^	0.976	0.582 ± 0.054
F	29.48 ± 2.68^i^	0.60 ± 0.016^e^	0.998	0.738 ± 0.036
G	43.80 ± 2.42^h^	0.63 ± 0.024^d^	0.987	0.690 ± 0.075
H	11.32 ± 1.32^jk^	0.54 ± 0.029^f^	0.996	0.782 ± 0.067
I	9.13 ± 1.43^k^	0.72 ± 0.017^b^	0.997	0.816 ± 0.038
J	282.38 ± 9.90^d^	0.28 ± 0.021^k^	0.971	0.692 ± 0.085
K	52.57 ± 4.95^g^	0.63 ± 0.012^d^	0.993	0.724 ± 0.076
L	33.98 ± 4.22^i^	0.59 ± 0.024^e^	0.992	0.692 ± 0.038
M	65.23 ± 5.39^e^	0.43 ± 0.017^g^	0.986	0.734 ± 0.102
N	57.78 ± 5.32^f^	0.62 ± 0.026^d^	0.950	0.698 ± 0.092
O	12.00 ± 1.98^jk^	0.71 ± 0.029^bc^	0.999	0.769 ± 0.066
P	16.21 ± 2.70^j^	0.58 ± 0.021^e^	0.987	0.805 ± 0.074

Similar letters in each column indicate no significant difference between treatments (*p* > .05).

## 4 CONCLUSION

Sensory evaluation definitely plays an important role in new food production and formulation in industry. According to the mentioned functional properties of pistachio oil, spreads that had solid fat replaced by nut oils are considered a functional food. These functional foods must be optimized in terms of texture, taste, and flavor. When evaluation of much of sensorial descriptors must be done (five or more), PCA is one of the most suitable tests for evaluating them because it shows the distribution of the components and different formulations well. Using PCA, plot 16 formulation was divided into four groups such that each of them had a special sensorial descriptor. PO, XG, DMG, and CB were the original variables that constitute different formulations and all of them had a significant effect on different textural attributes. The optimal formulation for producing chocolate with textural properties with maximum desirability was 15% PO, 7.5% CB, 0.3% XG, and 1% DMG.

## AUTHOR CONTRIBUTIONS

**Morad Mousazadeh:** Methodology (equal); project administration (equal). **Mohammad Mousavi:** Supervision (equal); validation (equal). **Zahra Emam‐Djomeh:** Resources (equal); writing – review and editing (equal). **Salar Ali Ahmed:** Formal analysis (equal). **Mehri Hadinezhad:** Conceptualization (equal); visualization (equal). **Hamed Hassanzadeh:** Software (equal); writing – review and editing (equal).

## FUNDING INFORMATION

The author(s) received no financial support for the research, authorship, and/or publication of this article.

## CONFLICT OF INTEREST STATEMENT

It is hereby confirmed that there are no known conflicts of interest related to this publication and that there was no significant financial support for this project.

## Data Availability

The data that support the findings of this study are available from the corresponding author upon reasonable request.

## References

[fsn33637-bib-0001] Afoakwa, E. O. , Paterson, A. , & Folwer, M. (2007). Factors influencing rheological and textural qualities in chocolate‐a review. Trends in Food Science and Technology, 18(6), 290–298.

[fsn33637-bib-0002] Alasalvar, C. , & Shahidi, F. (2008). Tree nuts: Composition, phytochemicals, and health effects. Boca Raton CRC press.

[fsn33637-bib-0003] Aryee, A. N. , & Boye, J. I. (2014). Current and emerging trends in the formulation and manufacture of nutraceuticals and functional food products. In J. I. Boye (Ed.), Nutraceutical and functional food processing technology (pp. 1–63). Wiley.

[fsn33637-bib-0004] Beckett, S. T. (1999). Industrial chocolate manufacture and use (3th ed.). Blackwell Science.

[fsn33637-bib-0005] Beckett, S. T. (2000a). In the science of chocolate. The Royal Society of Chemistry.

[fsn33637-bib-0006] Beckett, S. T. (2000b). The science of chocolate. Royal Society of Chemistry.

[fsn33637-bib-0007] Bemer, H. L. , Limbaugh, M. , Cramer, E. D. , Harper, W. J. , & Maleky, F. (2016). Vegetable organogels incorporation in cream cheese products. Food Research International, 85, 67–75. 29544854 10.1016/j.foodres.2016.04.016

[fsn33637-bib-0008] Bonczar, G. , Wszolek, M. , & Siuta, A. (2002). The effects of certain factors on the properties of yoghurt made from ewe's milk. Food Chemistry, 79, 85–91.

[fsn33637-bib-0009] Boualem, S. A. , Karci, H. , Kafkas, S. , Elouissi, A. , & Nogay, G. (2022). Quality index based on fatty acids for Syrian pistachio cultivars (Pistacia vera L.) grown in Mascara (north‐west of Algeria). Acta Agriculturae Slovenica, 118(4), 1–8.

[fsn33637-bib-0011] Brunello, N. , Mcgauley, S. E. , & Marangoni, A. (2003). Mechanical properties of cocoa butter in relation to its crystallization behavior and microstructure. LWT – Food Sci. Technology, 36, 525–532.

[fsn33637-bib-0012] Casas, J. A. , & Garcia‐Ochoa, F. (1999). Viscosity of solutions of xanthan/locust bean gum mixtures. Journal of the Science of Food and Agriculture, 79, 25–31.

[fsn33637-bib-0013] Cheraghali, A. M. , & Yazdanpanah, H. (2010). Interventions to control aflatoxin contamination in pistachio nuts: Iran experience. Journal of Food Safety, 30, 382–397.

[fsn33637-bib-0014] Chu, C. A. , & Resurreccion, A. V. A. (1991). Optimization of a chocolate peanut spread using response surface methodology (RSM). Journal of Sensory Studies, 19(3), 237–260.

[fsn33637-bib-0015] Chu, C. A. , & Resurreccion, A. V. A. (2005). Sensory profiling and characterization of chocolate peanut spread using response surface methodology. Journal of Sensory Studies, 20(3), 243–274.

[fsn33637-bib-0016] De, L. C. (2020). Edible seeds and nuts in human diet for immunity development. International Journal of Recent Scientific Research, 6(11), 38877–38881.

[fsn33637-bib-0017] Deshpande, R. P. , Chinnan, M. S. , & Mcwatters, K. H. (2005). Nutritional, physical and sensory characteristics of various chocolate‐flavored peanut–soy beverage formulations. Journal of Sensory Studies, 20(2), 130–146.

[fsn33637-bib-0018] D'Evoli, L. , Lucarini, M. , Gabrielli, P. , Aguzzi, A. , & Lombardi‐Boccia, G. (2015). Nutritional value of Italian pistachios from Bronte (Pistacia vera, L.), their nutrients, bioactive compounds and antioxidant activity. Food and Nutrition Sciences, 6(14), 1267–1276.

[fsn33637-bib-0020] Full, N. A. , Yella Reddy, S. , Dimick, P. S. , & Ziegler, G. R. (1996). Physical and sensory properties of milk chocolate formulated with anhydrous milk fat fractions. Journal of Food Science, 61, 1068–1072.

[fsn33637-bib-0023] Gharibzahedi, S. M. T. , Mousavi, S. M. , Hamedi, M. , & Ghasemlou, M. (2012). Response surface modeling for optimization of formulation variables and physical stability assessment of walnut oil‐in‐water beverage emulsions. Food Hydrocolloids, 26, 293–301.

[fsn33637-bib-0024] Gharibzahedi, S. M. T. , Mousavi, S. M. , Hamedi, M. , & Khodaiyan, F. (2011). Application of response surface modeling to optimize critical structural components of walnut–beverage emulsion with respect to analysis of the physicochemical aspects. Food and Bioprocess Technology, 6, 456–469. 10.1007/s11947-011-0763-8

[fsn33637-bib-0025] Gharibzahedi, S. M. T. , Mousavi, S. M. , Khodaiyan, F. , & Hamedi, M. (2012). Optimization and characterization of walnut beverage emulsions in relation to their composition and structure. International Journal of Biological Macromolecules, 50, 376–384. 22210526 10.1016/j.ijbiomac.2011.12.008

[fsn33637-bib-0026] Glibowski, P. , Zarzycki, P. , & Krzepkowska, M. (2008). The rheological and instrumental textural properties of selected table fats. International Journal of Food Properties, 11, 678–686.

[fsn33637-bib-0027] Gonçalves, E. V. , & Lannes, S. C. S. (2010). Chocolate rheology. Ciência e Tecnologia de Alimentos, 30, 845–851.

[fsn33637-bib-0028] González‐Tomás, L. , & Costell, L. (2006). Sensory evaluation of vanilla‐dairy desserts by repertory grid method and free choice profile. Journal of Sensory Studies, 21(1), 20–33.

[fsn33637-bib-0029] Hinds, M. J. , Chinnan, M. S. , & Beuchat, L. R. (1994). Unhydrogenated palm oil as a stabilizer for peanut butter. Journal of Food Science, 59(4), 816–820.

[fsn33637-bib-0030] Hough, G. , & Garitta, L. (2012). Methodology for sensory shelf‐life estimation: A review. Journal of Sensory Studies, 27(3), 137–147.

[fsn33637-bib-0031] Imram, N. (1999). Visual cues identified by a visual profile panel in the evaluation of chilled dairy desserts. Journal of Sensory Studies, 14(3), 369–386.

[fsn33637-bib-0032] Khaleghi Yazdi, T. , Afshar Mogaddam, M. R. , Mousazadeh, M. , Monajjemzadeh, F. , Tamizi, E. , & Nemati, M. (2021). Differential scanning calorimetry and fatty acid composition analysis of chocolates marketed in Iran as an alternative method for identification of cocoa butter adulteration. Journal of AOAC International, 104(6), 1479–1484. 34180991 10.1093/jaoacint/qsab086

[fsn33637-bib-0033] Kim, B. H. , Shewfelt, R. L. , Lee, H. , & Akoh, C. C. (2005). Sensory evaluation of butterfat‐vegetable oil blend spread prepared with structured lipid containing canola oil and caprylic acid. Journal of Food Science, 70(7), 406–412.

[fsn33637-bib-0035] Liu, J. , Zhou, H. , Tan, Y. , Mundo, J. L. M. , & McClements, D. J. (2021). Comparison of plant‐based emulsifier performance in water‐in‐oil‐in‐water emulsions: Soy protein isolate, pectin and gum Arabic. Journal of Food Engineering, 307, 110625.

[fsn33637-bib-0036] Marangoni, A. G. , & Mcgauley, S. E. (2003). Relationship between crystallization behavior and structure in cocoa butter. Crystal Growth & Design, 3, 95–108.

[fsn33637-bib-0037] Mewis, J. (1979). Thixotropy—A general review. Journal of Non‐Newtonian Fluid Mechanics, 6, 1–20.

[fsn33637-bib-0038] Morzel, M. , Heapes, M. M. , Reville, W. J. , & Arendt, E. K. (2000). Textural and ultrastructural changes during processing and storage of lightly preserved salmon (*Salmo salar*) products. Journal of the Science of Food and Agriculture, 82, 1691–1697.

[fsn33637-bib-0039] Mousazadeh, M. , Mousavi, M. , Emam‐Djomeh, Z. , Hadinezhad, M. , & Gharibzahedi, S. M. T. (2014). Formulation optimization of pistachio oil spreads by characterization of the instrumental textural attributes. International Journal of Food Properties, 17(6), 1355–1368.

[fsn33637-bib-0040] Mousazadeh, M. , Mousavi, S. M. , Emam‐Djomeh, Z. , Hadinezhad, M. , & Rahmati, N. (2013). Stability and dynamic rheological characterization of spread developed based on pistachio oil. International Journal of Biological Macromolecules, 56, 133–139. 23411037 10.1016/j.ijbiomac.2013.02.001

[fsn33637-bib-0041] Narine, S. S. , & Marangoni, A. G. (1999). Relating structure of fat crystal networks to mechanical properties: A review. Food Research International, 32(4), 227–248.

[fsn33637-bib-0042] Nattress, L. A. , Ziegler, G. R. , Hollender, R. , & Peterson, D. G. (2004). Influence of hazelnut paste on the sensory properties and shelf‐life of dark chocolate. Journal of Sensory Studies, 19, 133–148.

[fsn33637-bib-0043] Pareyt, B. , Talhaoui, F. , Kerckhofs, G. , Brijs, K. , Goesaert, H. , Wevers, M. , & Delcour, J. A. (2009). The role of sugar and fat in sugar‐snap cookies: Structural and textural properties. Journal of Food Engineering, 90, 400–408.

[fsn33637-bib-0044] Radočaj, O. , Dimić, E. , Diosady, L. L. , & Vujasinović, V. (2011). Optimizing the texture attributes of a fat‐based spread using instrumental measurements. Journal of Texture Studies, 42, 394–403.

[fsn33637-bib-0047] Shakerardekani, A. , Karim, R. , Ghazali, H. M. , & Chin, N. L. (2013). Textural, rheological and sensory properties and oxidative stability of nut spreads—A review. International Journal of Molecular Sciences, 14(2), 4223–4241. 23429239 10.3390/ijms14024223 PMC3588096

[fsn33637-bib-0048] Shakerardekani, A. , Karim, R. , Ghazali, H. M. , & Chin, N. L. (2015). Oxidative stability of pistachio (Pistacia vera L.) paste and spreads. Journal of the American Oil Chemists' Society, 92(7), 1015–1021.

[fsn33637-bib-0049] Shakerardekani, A. , Karim, R. , & Mirdamadiha, F. (2012). The effect of sorting on aflatoxin reduction of pistachio nuts. J. Food. Agriculture and Environment, 10, 459–461.

[fsn33637-bib-0050] Sharif, M. K. , Butt, M. S. , Sharif, H. R. , & Nasir, M. (2017). Sensory evaluation and consumer acceptability. In Handbook of food science and technology (pp. 361–386). University of Agriculture.

[fsn33637-bib-0051] Smith, G. L. (1988). Statistical analysis of sensory data. In J. R. Piggott (Ed.), Sensory Analysis of Foods (pp. 335–379). Elsevier Science Publishers.

[fsn33637-bib-0052] Soares Mateus, A. R. , Barros, S. , Pena, A. , & Sanches Silva, A. (2021). Mycotoxins in pistachios (pistacia vera L.): Methods for determination, occurrence, decontamination. Toxins, 13(10), 682. 34678975 10.3390/toxins13100682 PMC8538126

[fsn33637-bib-0053] Steffe, J. F. (1996). Rheological methods in food process engineering. Freeman Press.

[fsn33637-bib-0054] Stone, H. , & Sidel, J. L. (1993). Descriptive analysis. In H. Stone & J. L. Sidel (Eds.), Sensory evaluation practices (2nd ed.). Academic Press.

[fsn33637-bib-0057] Tanaka, L. , Tanaka, K. , Yamato, S. , Ueno, S. , & Sato, K. (2009). Microbeam X‐ray diffraction study of granular crystals formed in water‐in‐oil emulsion. Food Biophysics, 4, 331–339.

[fsn33637-bib-0058] Tomaino, A. , Martorana, M. , Arcoraci, T. , Monteleone, D. , Giovinazzo, C. , & Saija, A. (2010). Antioxidant activity and phenolic profile of pistachio (*Pistacia vera* L., variety Bronte) seeds and skins. Biochimie, 92(9), 1115–1122. 20388531 10.1016/j.biochi.2010.03.027

[fsn33637-bib-0059] Vahedi, H. , & Mousazadeh, M. (2016). The effect of using stevia and agave nectar as a substitute for sucrose on physical, chemical, rheological, and sensory properties of dark chocolate. Der Pharmacia Lettre, 8(15), 194–201.

[fsn33637-bib-0060] Veland, J. O. , & Torrissen, O. J. (1999). The texture of Atlantic salmon (*Salmo salar*) muscle as measured instrumentally using TPA and Warner‐Bratzler shear test. Journal of the Science of Food and Agriculture, 79, 1737–1746.

[fsn33637-bib-0061] Vereecken, J. , Foubert, I. , Meeussen, W. , Lesaffer, A. , & Dewettinck, K. (2009). Fat structuring with partial triacylglycerols: Effect on solid fat profiles. European Journal of Lipid Science and Technology, 110, 259–272.

[fsn33637-bib-0062] Wille, R. L. , & Lutton, E. S. (1966). Polymorphism of cocoa butter. Journal of the American Oil Chemists' Society, 43, 491–496. 5945032 10.1007/BF02641273

[fsn33637-bib-0065] Yahia, E. M. , García‐Solís, P. , & Celis, M. E. M. (2019). Contribution of fruits and vegetables to human nutrition and health. In E. M. Yahia & P. García‐Solís (Eds.), Postharvest physiology and biochemistry of fruits and vegetables (pp. 19–45). Woodhead Publishing.

[fsn33637-bib-0063] Yang, J. , Liu, R. H. , & Halim, L. (2009). Antioxidant and antiproliferative activities of common edible nut seeds. LWT ‐ Food Science and Technology, 42, 1–8.

[fsn33637-bib-0064] Yeh, J. Y. , Phillips, R. D. , & Hung, Y. C. (2003). Optimizing protein‐ and vitamin‐fortified peanut spreads containing soybean or milk powder. Journal of Food Quality, 26(3), 243–256.

